# Examining explainable artificial intelligence in TNM staging with PET-CT: a user-centred observation study

**DOI:** 10.1007/s11547-026-02226-9

**Published:** 2026-07-13

**Authors:** Charlie Baskerville, Julien M. Y. Willaime, Vineet Prakash, Tamir Ali, Shaheel Bhuva, Gordon Ellul, Russell Frood, Sachin Kamat, Sudeshna Maitra, David Rosewarne, Andrew Scarsbrook, Peter D. Strouhal, Amir Zarei, Kevin Wells

**Affiliations:** 1 Centre for Vision, Speech and Signal Processing, The University of Surrey, Guildford, UK; 2 Mirada Medical Ltd., Oxford, UK; 3 Royal Surrey NHS Foundation Trust, Guildford, UK; 4 Alliance Medical Ltd., Warwick, UK; 5 The Newcastle Upon Tyne Hospitals NHS Foundation Trust, Newcastle Upon Tyne, UK; 6 Oxford University Hospitals NHS Foundation Trust, Oxford, UK; 7 East Kent Hospitals University NHS Foundation Trust, Canterbury, UK; 8 Leeds Teaching Hospitals NHS Trust, Leeds, UK; 9 Faculty of Medicine and Health, University of Leeds, Leeds, UK; 10 King’s College London NHS Foundation Trust, London, UK; 11 The Royal Wolverhampton NHS Trust, Wolverhampton, UK

**Keywords:** PET-CT, AI, XAI, Implementation science, Clinical decision support systems, Radiology

## Abstract

**Purpose:**

Artificial intelligence (AI) is increasingly proposed as a solution to improve efficiency in radiology and nuclear medicine, particularly in the context of workforce shortages. However, adoption of AI-based clinical decision support systems (AI-CDSS) remains slow, due to limited model transparency. Explainable AI (XAI) may improve clinician acceptance by supporting oversight and trust. This study evaluated the impact of different XAI explanation types on radiologists’ willingness to adopt AI systems.

**Methods:**

Ten nuclear medicine radiologists from eight UK institutions performed lung cancer TNM staging using whole-body PET/CT scans supported by a simulated AI-CDSS. Three explanation approaches were assessed: input feature attribution, high-level concept explanations and global algorithmic transparency. Adoption likelihood and explanation usefulness were rated using Likert scales and analysed with nonparametric sign tests. Semi-structured interviews were additionally analysed through thematic evaluation supported by large language model-assisted coding with human verification.

**Results:**

All explanation approaches significantly increased radiologists’ willingness to adopt the AI system compared to a black-box model (*p* < 0.05). Explanations were consistently considered useful in enabling participants to confirm or challenge AI staging recommendations (*p* < 0.001). Qualitative findings highlighted the importance of clinical relevance, error detection and decision support value. A trade-off between explanation depth and usability was identified as a key factor influencing preferences.

**Conclusion:**

Incorporating XAI into nuclear medicine CDSS enhances radiologists’ acceptance and provides clinically meaningful information for oversight of AI recommendations, in accordance with the EU AI Act. These findings support the role of XAI in facilitating integration of AI tools into diagnostic workflows.

**Supplementary Information:**

The online version contains supplementary material available at https://doi.org/10.1007/s11547-026-02226-9.

## Introduction

Global staff shortages in radiology continue to raise concerns over the impact on patient care [1, 2]. The Royal College of Radiologists reported a 30% shortfall in UK radiologists in 2023, expected to rise in subsequent years [3]. 70% of clinical directors report this as a concern for safe and effective patient care [3]. Artificial Intelligence (AI) has been proposed to help alleviate workforce pressures and improve clinical workflow through decision support [4]. In nuclear medicine, applications include lesion detection, automatic staging and treatment response prediction [5–7].

Despite these capabilities, clinical adoption of AI tools remains limited, as high technical performance in research often fails to translate to clinical use [8, 9]. Beyond technical limitations, adoption barriers exist throughout the product lifecycle, ranging from logistical hurdles like staff training costs and system interoperability to critical end-user concerns regarding performance expectancy, independent validation and system transparency [10, 11]. These end-user reservations are substantial; in a recent workshop, 60% of radiologists cited concerns over accuracy and reliability as major barriers to implementation [12]. While some argue that transparency is overstated as a barrier, the lack of clarity regarding model logic, training data and decision processes is widely reported as a significant issue compromising user trust and preventing confident adoption [12, 13].

Regulatory requirements add further complexity. The EU AI Act mandates human oversight for high-risk AI, such as those used in health care, and gives users the right to disregard, override or reverse AI system outputs [14]. This aspect underlines the need for transparency in AI decision-making via explainable AI (XAI).

Lack of transparency, especially in complex models like neural networks, undermines both trust and human oversight [15, 16]. XAI seeks to improve understanding of AI decision-making through human-interpretable explanatory outputs [17]. However, inconsistencies in terminology and approaches complicate the nascent field [18]. This study adopts the following definitions shown in Table 1. Although the primary focus of this study is interpretability, the term ‘XAI’ is adopted throughout to align with established literature [16, 17].

**Table 1:** Definitions of key terms used in this study

Transparency	The visibility over the whole machine learning pipeline including training data, training process, model architecture and model interpretability. This is consistent with the’trustworthy AI’ literature and regulatory domains [19, 20]
Interpretability	The level to which a user can understand the internal structure or decision-making process of an AI system
Explainability	The degree to which the decision-making process of a complex ’black-box’ model can be determined

In addition to technical development, there is a growing need to understand XAI’s impact on users. Yet, only 22% of XAI studies include users, and just 23% of those involve domain experts [21]. This study focuses on expert user-based evaluation of different XAI approaches that could be adopted in PET-CT reporting.

XAI evaluation methods are typically either computer-centred or human-centred [22]. Task-based assessment by domain experts remains the gold standard for XAI evaluation; however, participant recruitment and task realism pose significant challenges [23]. In addition, XAI approaches may generate inaccurate explanations of AI model behaviour, potentially hindering fair evaluation across different levels of interpretability [22]. Several criteria guide XAI evaluation, including trust, usability and alignment with user knowledge. Nauta et al. identified the ‘Co-12’ criteria, which include contextual relevance, coherence with prior knowledge and explanation interactivity [21]. Additional evaluation dimensions include trust calibration, usability and human–AI collaborative performance [24]. Common XAI evaluation methods include think-aloud protocols [25], eye-tracking studies [26] and semi-structured interviews [27–29]. Notably, Bergquist et al. conducted semi-structured interviews with radiologists, managers and developers to explore adoption issues [29].

Most studies examine a single XAI method, precluding analysis of user preferences across different explanation types. One exception, Naiseh et al., used Likert scales to evaluate multiple XAI methods in drug prescription decisions [30]. The isolated focus of many XAI studies lack the granularity needed to assess stakeholder preferences across the broad spectrum of XAI techniques. While a number of XAI taxonomies focus on technical dimensions such as method, scope and application, absent from the current literature is an agreed taxonomy which encompasses the different stakeholders [17, 31, 32]. Such stakeholders include radiologists, AI developers, regulatory bodies and patients, possessing different domain knowledge and objectives [33]. Saboury et al. regard stakeholder consideration is key for trustworthy AI systems [34]. With the anticipated inclusion of explainability requirements in AI legislation and the current limited understanding of stakeholder XAI preferences, addressing this stakeholder consideration gap becomes increasingly important [3]. The lack of user-centred validation in image-based XAI applications in radiology is the primary motivation for this work, given the central role of image interpretation and the increasing use of AI in such workflows.

Thus, the primary aim of this study was to investigate the impact of XAI with respect to routine clinical adoption and the usefulness of XAI-based clinical decision support systems (XAI-CDSS). Assessment of a wide range of XAI was based on a novel stakeholder-centric framework—The hierarchy of AI interpretability. A task-based user study was conducted utilising lung cancer PET-CT staging as the primary clinical task, wherein radiologists actively assessed the optimal levels of interpretability. This is the first such study to examine and compare a variety of XAI methods with radiologists as key stakeholders.

## Materials and methods

### Hierarchy of AI interpretability

**Fig. 1:**
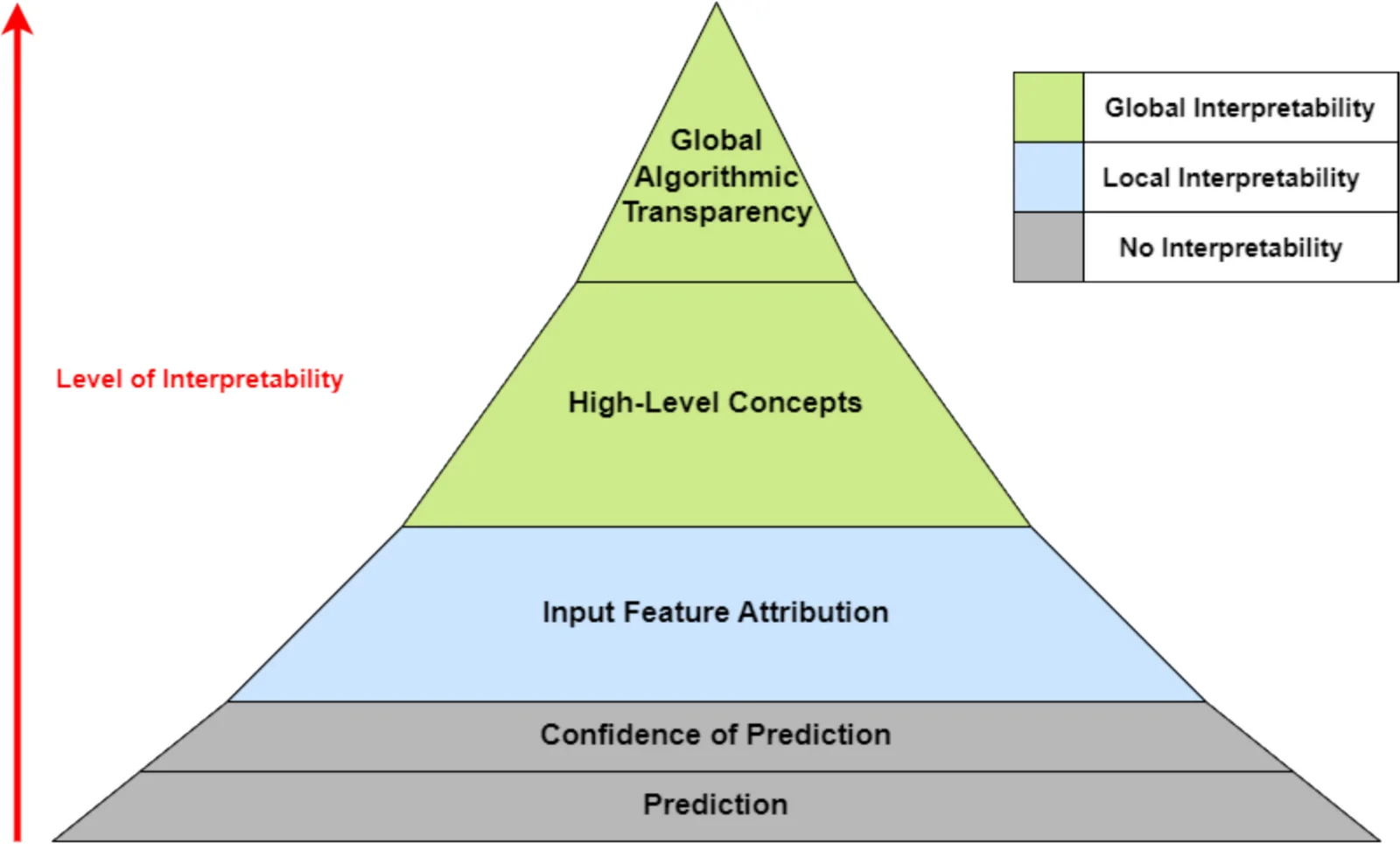
A Hierarchy of AI interpretability. The depth of explanation associated with the AI decision-making process increases with higher levels of the hierarchy. Interpretability levels are split into two types: local interpretability considers a single prediction, and global interpretability covers a set of predictions

Inspired by Maslow’s hierarchy of needs [35], this study proposes a new structure for which the degree of model interpretability (explanation depth) is expressed as a layered hierarchy. This novel structure formalises interpretability requirements against stakeholder needs (see Fig. 1), allowing for stakeholder comparison. To prioritise stakeholder needs, this hierarchy is abstracted from technical details such as methodology and format.

‘Scope’ is defined as the primary axis of the hierarchy, distinguishing between local methods that explain individual instances (e.g. ‘Why this patient?’) and global methods that explain overarching system logic [32]. However, while this distinction remains a useful tool for evaluating stakeholder needs, testing only local and global scope insufficiently differentiates the numerous XAI approaches currently available in the literature. From a user’s perspective, explanation scope is synonymous with explanation depth, specifically global scope explanations provide ‘deeper’ model understanding than local scope explanations. Surveying the XAI literature with a focus on explanation depth demonstrates clear hierarchical levels representing fundamental distinctions in explanation depth offered by current approaches [12, 15]. Such levels are intentionally broad to facilitate effective discrimination by stakeholders.

The hierarchy’s base layer (prediction, see Fig. 1) represents model predictions and associated confidence levels, offering minimal interpretability. While confidence scores provide some model insight, their inclusion is often an implementation choice rather than architectural decisions for interpretability. Input feature attributions (IFA) identify the most influential features driving predictions such as Gradient-weighted Class Activation Maps (Grad-CAM) visualisations [36]. These explanations operate at a local scope, focusing on specific input instances. In contrast, global scope interpretability covers a set of predictions which reveals information about the general AI decision-making process. This includes high-level concepts (HLC) learned during training, such as training data clustering patterns [37]. The hierarchy culminates in the global algorithmic transparency (GAT) layer, where model components are visible and can be decomposed and simulated independently, as exemplified by ‘white-box’ models [38].

Using this framework, stakeholder (in this case radiologist) needs can be investigated in a controlled study. Multiple levels of the hierarchy were tested for the following hypotheses:

*Adoption* Radiologists are more likely to adopt an AI-CDSS featuring interpretability level [IFA, HLC and GAT] into routine clinical practice compared to a black-box AI-CDSS.

*Usefulness* Which interpretability levels [IFA, HLC and GAT] provide radiologists with useful information from which they can more confidently accept or reject an AI recommendation?

### Study design

#### Task-based interviews

**Fig. 2:**
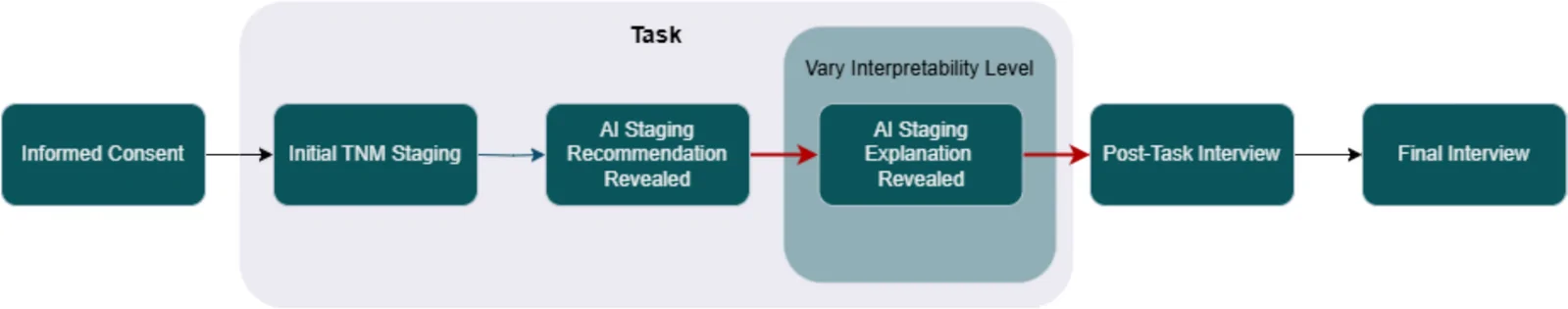
Session structure of the task-based interview with radiologists. Red arrows represent opportunities for the radiologists to amend patient stage and confidence score

Ten radiologists performed lung cancer tumour-node-metastasis (TNM) staging tasks on whole-body [18F] fluorodeoxyglucose (FDG) PET-CT scans utilising image data from the publicly available University Hospital Tübingen dataset [39]. Mirada XD version 3.6.11 diagnostic software (Mirada Medical Ltd., Oxford, UK) presented the scans integrated with mock AI staging recommendations and explanations. The structured assessment protocol is shown in Fig. 2. Following an initial staging, radiologists were given two sequential opportunities to revise their assessments based first on an AI recommendation and subsequently on an AI explanation [40]. During each revision phase, participants provided Likert scale ratings of agreement with the AI recommendations. Explanations were presented from a lower to a higher level of interpretability, preventing information ‘leaking’ from higher levels.

Each radiologist evaluated three cases selected from a pool of ten, with one case assigned to each interpretability level. Following each case, participants completed a semi-structured interview, provided in full in Online Resource Section 1, specifically designed to evaluate adoption, trust, usability and satisfaction [22, 41]. During these post-task evaluations, radiologists were asked to discuss the plausibility and overall quality of the provided explanations, identify potential AI limitations and qualitatively compare their reporting experience against a standard black-box system. To enable quantitative analysis, five-point Likert scales were integrated to measure the usefulness of the explanation for staging agreement, the overall impact on user confidence and the likelihood of adopting the explainable system into clinical practice. Upon completing all cases, a final interview was conducted where participants ranked the explanation types and reflected on whether their perceptions regarding the clinical adoption of AI had changed during the session. All interviews were conducted and recorded via Microsoft Teams, employing an evaluation methodology prevalent in explainable AI studies to elicit comprehensive yet focused responses [27–29].

**Fig. 3:**
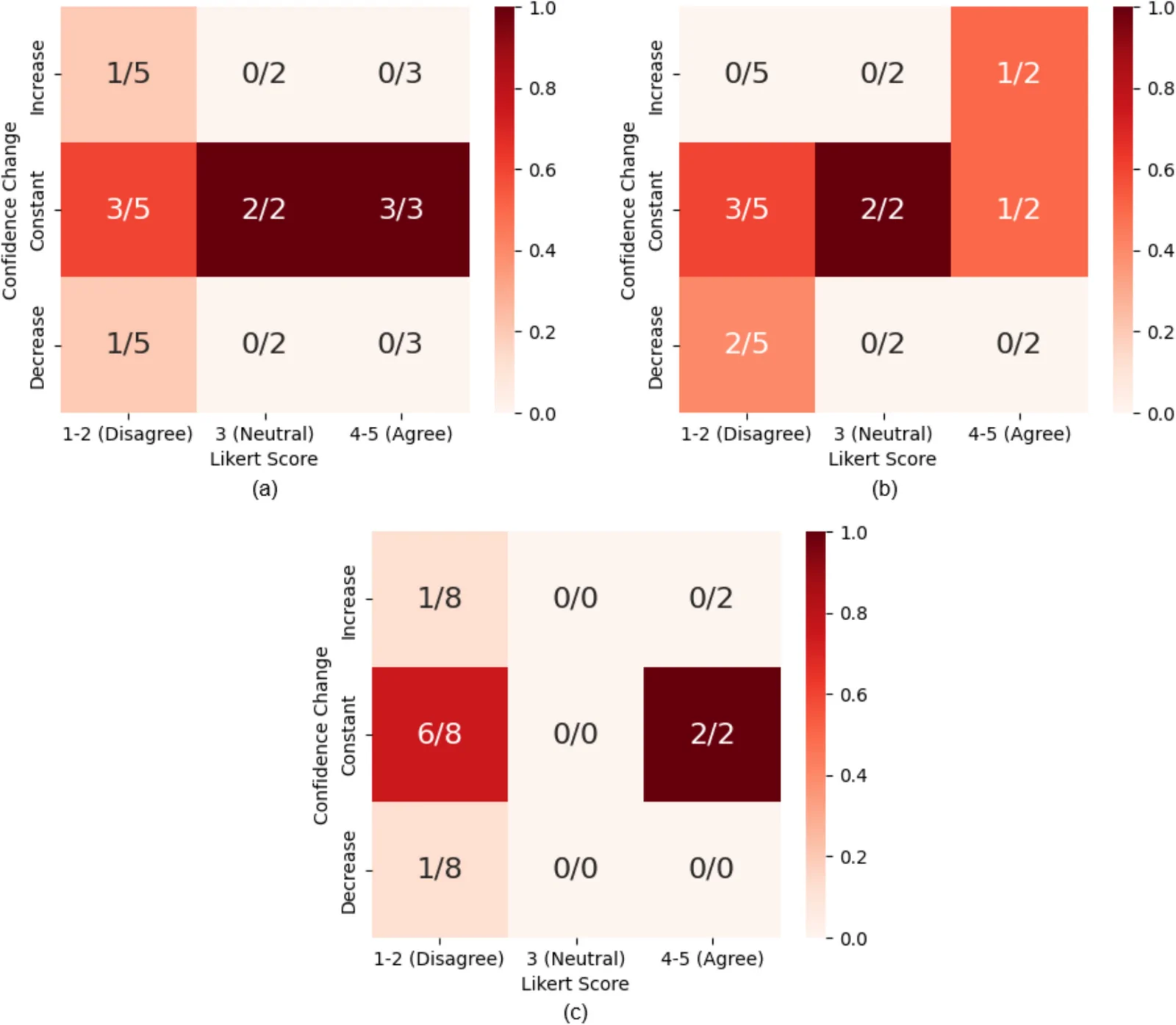
Stage confidence changes as a result of explanations (**a** input feature attributions, **b** high-level concepts, **c** global algorithmic transparency) confounded with human–AI agreement. One participant omitted from **b** due to interview error

**Fig. 4:**
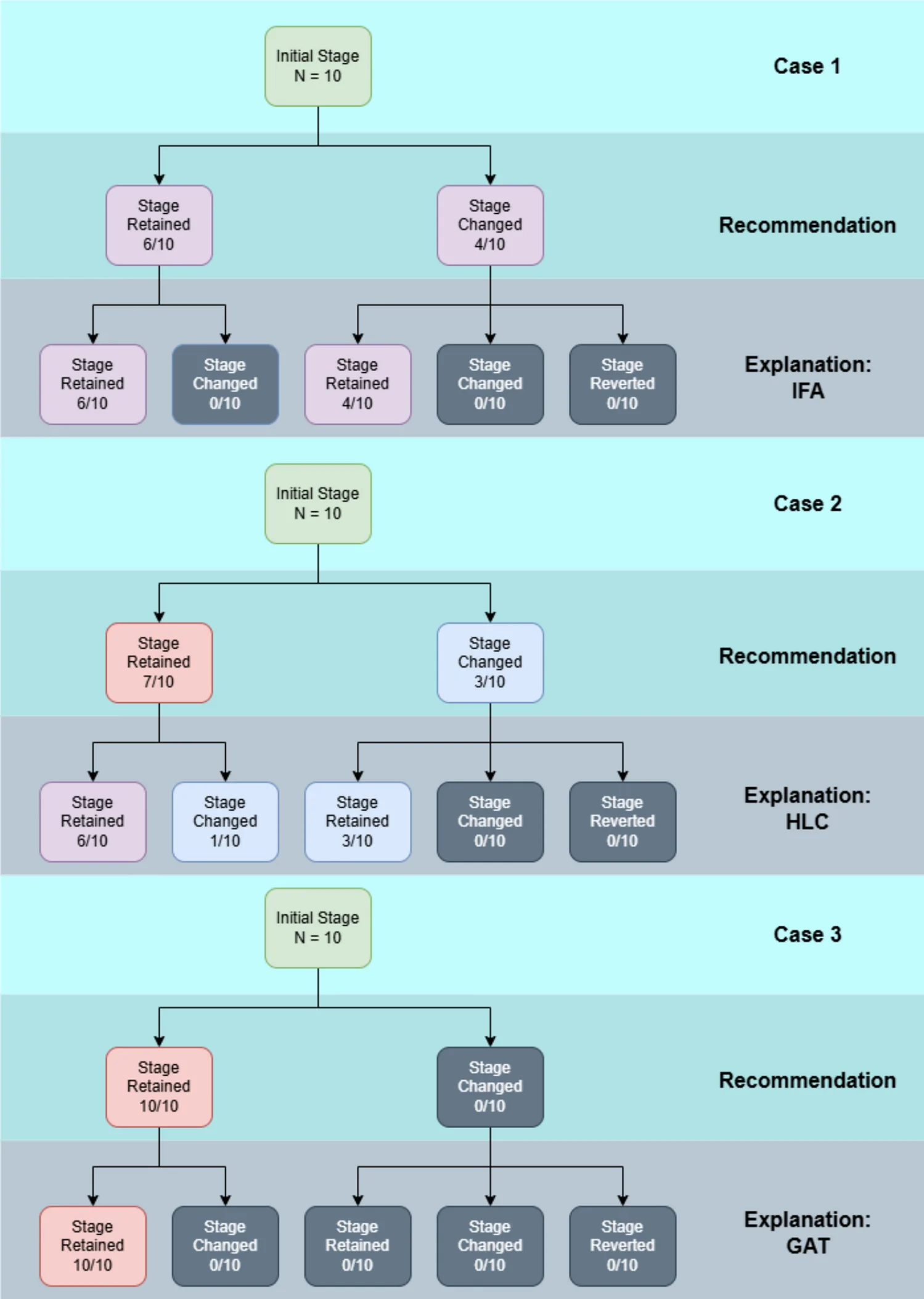
Diagram outlining decision/revision paths of radiologists that could retain/change the patient’s stage after seeing AI staging recommendation and explanation

**Fig. 5:**
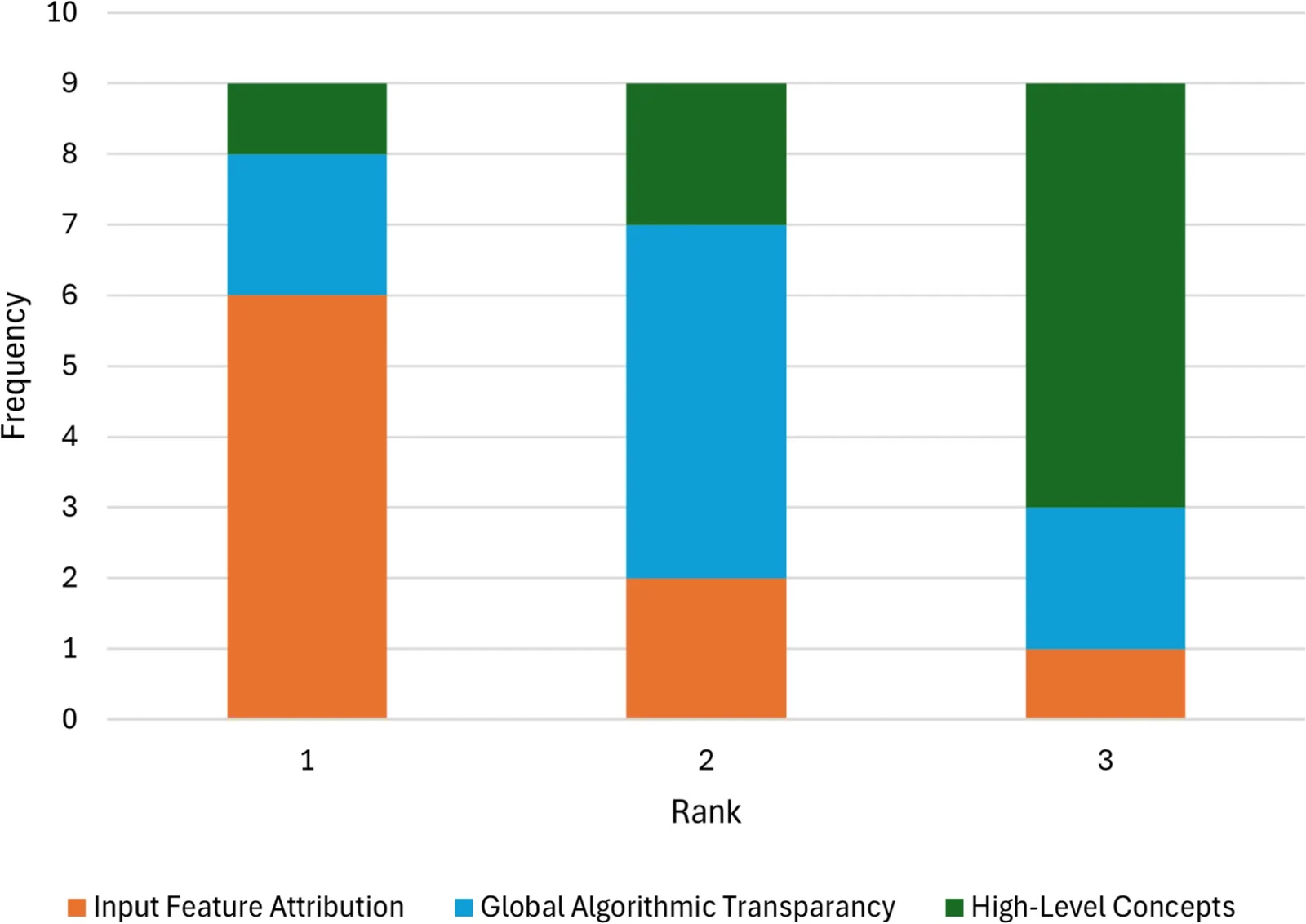
Preference rankings frequency chart of AI interpretability levels. Rank 1—favourite, rank 3—least favourite. One participant omitted due to interview error. This demonstrates a preference for input feature attribution with high-level concepts being the least favoured approach

To ensure fair evaluation without the bias of technically inaccurate XAI outputs, mock recommendations and explanations were derived from authentic radiological reporting by VP (consultant radiologist) [22]. IFA, HLC and GAT explanations employed heatmaps, feature plots and decision trees, respectively, as explanation categories identified by Nauta et al. [21]. To deliberately engineer human–AI disagreement, one staging recommendation was strategically modified for each participant. All content was clinically verified by VP and embedded within Mirada XD, with visibility controlled by the interviewer. Examples are shown in Online Resource Section 2 Figs. 1–5.

**Fig. 6:**
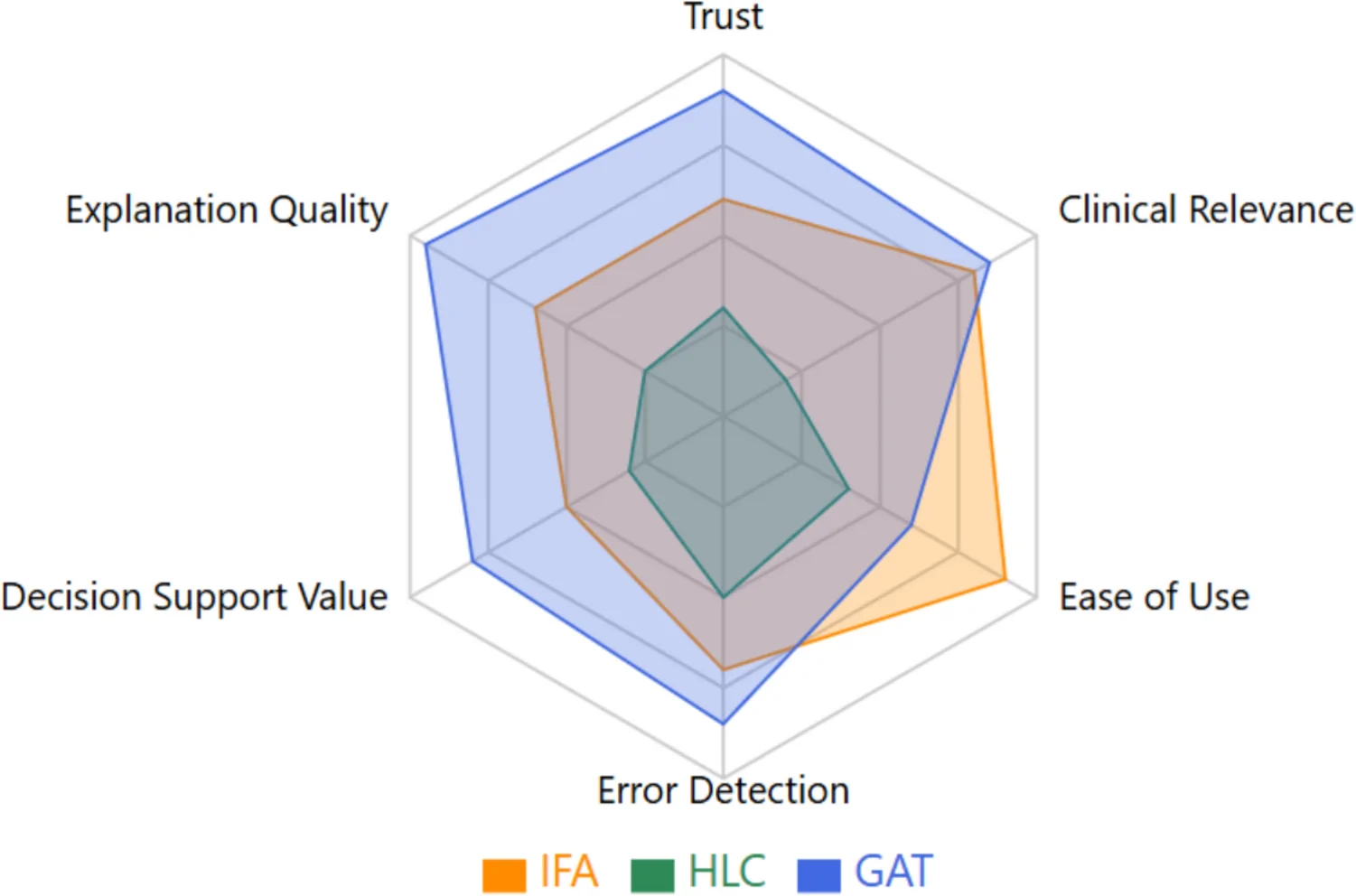
Radar chart comparing input feature attribution (IFA), high-level concepts (HLC) and global algorithmic transparency (GAT) with respect to key themes extracted from post-task interviews. Diagram generated using Anthropic’s Claude 3.5-sonnet LLM and verified for correctness

Ten expert radiologists (TA, SB, GE, RF, SK, SM, DR, AS, PDS and AZ) from eight UK institutions participated, averaging 14.7 years of experience (range: 5–25 years) (see Online Resource Section 3 Fig. 6). All actively performed TNM staging with PET-CT, four of whom had prior AI research experience.

### Analysis

Likert scores (1–5) were analysed with one-tailed sign tests (IBM SPSS Statistics 29) to evaluate the hypotheses outlined in Table 2. Sign tests measured whether scores were positive or negative compared to a baseline. This baseline represented a ‘black-box’ system, an AI that suggests a TNM stage without an explanation.

**Table 2:** Hypotheses to study the impact on stakeholder considerations of explanations of input feature attributions (IFA), high-level concepts (HLC) and global algorithmic transparency (GAT)

Stakeholder Consideration	Hypothesis Tested
Adoption	Radiologists are more likely to adopt an AI-CDSS featuring interpretability level [IFA, HLC and GAT] into routine clinical practice compared to a black-box AI-CDSS. (H1-A) Note: null hypothesis is referred to as H0-A
Information Usefulness	The XAI types [IFA, HLC and GAT] do provide useful information by which a radiologists can accept or reject an AI staging recommendation. (H1-B) Note: null hypothesis is referred to as H0-B

Following transcript verification and organisation, interview analysis was conducted with Anthropic’s Claude 3.5-sonnet large language model (LLM), guided by established thematic analysis principles [42, 43]. This approach was selected based on evidence supporting LLM effectiveness in deductive coding and healthcare interview interpretation [44–46]. Clearly defined initial themes were identified by a human coder to minimise LLM misinterpretation. Prompt engineering was iteratively refined to enhance analysis accuracy, informed by Zhang et al. findings [44]. All findings were verified using supporting quotes, and final judgement rested with the human coder to avoid LLM hallucination risks [47].

## Results

### Quantitative analysis

Most participants were more likely to adopt XAI across explanation types over a black-box system (see Online Resource Section 3 Fig. 7). For each explanation type, one participant maintained equivalent adoption likelihood to black-box systems, while a single participant showed significantly reduced adoption preference for GAT explanations.

As shown in Table 3, one-tailed sign tests demonstrated significantly higher adoption likelihood for IFA (median = 5.0, *p* = 0.001), HLC (median = 4.5, *p* < 0.005) and GAT explanations (median = 4.0, *p* < 0.05) compared to black-box systems, supporting H1-A.

**Table 3:** Results of the one-tailed nonparametric sign test for XAI-CDSS adoption compared with a black-box AI-CDSS (hypothesised median Likert score 3)

XAI type	Sample Size	Median Likert Score	Positive Differences	Negative Differences	Ties	*p*-value
Input Feature Attribution	10	5.0	10	0	0	= 0.001
High-Level Concepts	10	4.5	9	0	1	< 0.005
Global Algorithmic Transparency	10	4.0	8	1	1	< 0.05

Usefulness Likert scores (shown in Online Resource Section 3 Fig. 8) demonstrated strong concentration around ‘very useful’ for GAT explanations, while IFA responses clustered in the 3–5 (overall positive) range, while HLC explanations exhibited high variance. One-tailed sign tests (shown in Table 4) confirmed significant usefulness for all explanation types: IFA (median = 3.5, *p* = 0.001), HLC (median = 4.0, *p* < 0.005) and GAT (median = 4.0, *p* < 0.005), supporting H1-B.

**Table 4:** Results of one-tailed nonparametric sign test for the explanation utility consideration (Likert scores compared to a hypothesised median of 1—XAI does not provide useful information)

XAI type	Sample Size	Median Likert Score	Positive Differences	Negative Differences	Ties	*p*-value
Input Feature Attribution	10	3.5	10	0	0	= 0.001
High-Level Concepts	10	4.0	9	0	1	< 0.005
Global Algorithmic Transparency	9*	4.0	8	0	1	< 0.005

*One participant omitted due to an interview error

AI system confidence impact varied significantly across all explanation types (see Online Resource Section 3 Fig. 9), persisting when data was grouped by human–AI staging agreement, though sample size limitations warrant consideration. As shown in Fig. 3, aggregated heatmaps illustrate changes in participants’ staging confidence post-explanation, grouped by initial AI recommendation agreement levels. Both IFA and GAT explanations only influenced confidence levels in cases of human–AI disagreement, whereas HLC explanations caused one participant to increase confidence in human–AI agreement. These findings suggest explanations had greater impact on confidence when initial human–AI disagreement existed.

Figure 4 illustrates the stage decision/revision pathways across explanation types (revision opportunities represented by the red arrows in Fig. 2). While IFA and HLC sections showed balanced stage changes following AI recommendations, all participants maintained initial staging during GAT assessment. Post-explanation, only one staging revision occurred (with HLC explanation), suggesting minimal impact of AI explanations on radiologists’ staging decisions.

Six radiologists ranked IFA as their top preference (Fig. 5). GAT was favoured by two and ranked second by five. HLC was the least preferred by most participants.

### Qualitative analysis

Key themes extracted from the interviews (listed in Online Resource Section 4 Table 1) formed the basis of comparison between interpretability levels, displayed in Fig. 6.

Lack of understanding about the AI decision-making process was frequently cited as a barrier to trust. IFA fostered basic trust through clarity, though insufficient for complex staging decisions. GAT generated the strongest trust by aligning with clinical reasoning, while HLC often undermined it due to the abstract format.

AI system confidence interacted with human–AI agreement and detection of AI system limitations. IFA increased confidence when aligning with radiologist’s observation and decreased when AI errors were exposed. HLC reduced confidence when global decision-making errors were detected, while GAT enhanced confidence in the AI system through their clinical familiarity.

In the interviews, explanation usefulness emerged as a prominent theme. GAT offered the greatest explanation depth, supporting transparency but also increasing cognitive load. HLC showed poor usefulness resulting from abstract visualisation and poor interpretability. IFA proved partially useful for particularly lesion detection, though lacking depth for staging rationale.

Error detection capabilities varied significantly across explanation types. IFA enabled local error detection through lesion verification but lacked the global evaluation of classification logic exhibited by GAT and HLC (exemplified by the oversimplified ‘radial distance from primary tumour’ criteria shown in Online Resource Section 2 Fig. 3). Participants often misattributed AI logic errors to poor explanation quality, particularly for HLC, decreasing AI system confidence and decision support value.

IFA was deemed both highly usable, collaborative and clinically relevant due to a direct imaging connection and alignment with routine lesion detection practices, offering optimal workflow integration. However, the clinical value was tempered by the limited insight into classification discrepancies. Conversely, GAT offered effective collaboration and detailed description of AI staging logic, increasing its clinical value. The detail potentially exceeded the requirements for straightforward cases, triggering usability and workflow integration concerns. These concerns were also observed in HLC which was generally deemed clinically irrelevant due to interpretability issues thus offering little clinical value. HLC showed potential for regulatory evaluation, a useful adjunct for vendors addressing the EU AI Act [14].

Transcript analysis revealed preference drivers: simplicity was preferred overall but was balanced with decision support value and clinical relevance. Though some found IFA depth limited, their ease of use resulted in them being preferred to other explanation types. GAT gained favour from four radiologists due to comprehensiveness and clinical intuition alignment, while HLC was least preferred due to interpretation difficulties. Figure 6 elucidates a key trade-off that emerged between explanation quality (specifically depth) and usability, contingent on clinical relevance. Radiological and AI experience levels showed no correlation with preferences, suggesting other determining factors.

## Discussion

This study examined a number of different XAI approaches of increasing depth—IFA, HLC and GAT applied to TNM staging with PET-CT scans. Interviews with radiologists testing simulated XAI-CDSS tools provided insights into explanation preferences and adoption likelihood.

Radiologists showed significantly higher adoption likelihood for all interpretability levels compared to black-box systems. Trust and system confidence were key drivers, supporting findings from Bergquist et al., who highlighted transparency and verification as crucial trust-related themes [29]. While Panigutti et al. reported limited trust impact in a different clinical setting, they still observed a preference for explanations—consistent with this study’s findings [40].

Clinical reasoning alignment proved crucial for establishing trust. HLC explanations’ poor perceived quality impeded assessment of alignment. Usability issues limited the integration of deeper explanations like HLC and GAT. Many participants felt the effort required to interpret HLC and GAT outweighed their decision support value. IFA, though less informative, was praised for ease of integration.

The secondary finding of this study was a preference trade-off which emerged between explanation depth and usability, with decision support value correlating with explanation depth but inversely affecting clinical integration ease, as demonstrated in Fig. 6. Participants advocated for configurable explanation depth which is then made adaptable to case complexity—this is a noteworthy finding for AI developers. Specifically, cases with greater perceived complexity, which are therefore more likely to generate a diversity of clinical interpretations, required deeper explanations. Configurability is further supported by Nauta et al., emphasising the importance of tailoring explanations to differing stakeholder expertise levels and perspectives [21]. This recommendation carries particular weight as findings may not generalise across different applications and stakeholders [24].

All explanation types proved useful for evaluating AI outputs, particularly during human–AI disagreement—an important area of investigation given the observed tendency of users to over-rely on XAI systems [30]. In such cases, deeper explanations aided error detection and decision arbitration. IFA explanations enabled lesion detection errors, while GAT explanations facilitated identification of subtle classification errors. Error detection capabilities are particularly relevant to seamless integration of AI into clinical workflows, enabling better understanding of how and when to defer/arbitrate between AI/human expertise as represented in the EU AI Act’s human oversight requirements [14]. Such capabilities could also alleviate performance expectancy concerns which represent important barriers to adoption [48, 49].

Confusion occurred when explanations that effectively revealed AI decision-making flaws often received negative feedback. This may partially explain HLC’s negative reception—despite being effective at revealing misalignment between learned concepts and clinical guidelines, they were criticised for lacking clinical relevance.

While small sample size limits generalisation, confidence heatmaps (Fig. 3) indicated greater confidence shifts during disagreement, warranting further investigation into collaborative decision-making. Despite frequent disagreements, explanations rarely altered staging decisions—potentially attributable to either insufficient AI interaction time for building trust or the deductive nature of TNM staging, where staging recommendations alone often revealed underlying lesion detection patterns.

IFA was the most preferred interpretability level, closely followed by GAT. IFA preference appeared to be driven by usability and clinical integration, while GAT advocates valued depth and decision support—further evidencing the explanations depth–usability trade-off. HLC ranked lowest, potentially due to unfamiliar formatting, though one participant favoured its balanced depth-to-usability ratio.

Study limitations primarily centred on restricted sample size and participant interaction time, with each radiologist evaluating only one case per interpretability level. It should be noted that the findings specifically address the task of staging in lung cancer using PET/CT data. Further studies would be needed across different cancer types and imaging modalities to better understand how these findings may generalise across difference applications. Limited interaction time may have hindered the potential for participants to develop trust with the black-box systems. Given that the explanation depth–usability trade-off varies by case complexity, future studies should assess multiple cases across interpretability levels. While the test environment was designed to approximate real-world conditions while enabling screen recording and interviewer interaction, it remained a somewhat artificial setting and represents a study limitation. Snowball sampling, although widely accepted and adopted across countless studies of this type, must also be recognised as a potential source of selection bias [50]. Additionally, while controlling explanation format across interpretability levels was methodologically sound, the chosen visual format for HLC explanations proved problematic, suggesting alternative presentation formats may yield alternative results. An example of alternative HLC formats is example-based explanations, specifically counterfactuals which were perceived to be the most comprehensible explanation type in a study by Naiseh et al. [30].

Future research with larger cohorts should validate the findings of this study and further investigate the identified explanation depth–usability trade-off across different stakeholder groups. Building on this study’s theoretical framework and radiologist feedback, subsequent investigations should evaluate real-world XAI implementations aligned with established stakeholder preferences.

## Conclusion

This study demonstrates that providing radiologists with explainable AI during lung cancer PET-CT staging significantly increases their willingness to adopt clinical decision support systems compared to baseline black-box models. The integration of artificial intelligence is significantly more likely to be embraced by radiologists when diagnostic systems provide transparent explanations rather than black-box recommendations. These explainable tools offer substantial clinical value by enabling end users to verify algorithmic logic, detect potential errors, and confidently arbitrate decisions during instances of human–AI disagreement. To optimise practical utility and achieve seamless workflow integration, future clinical decision support systems should feature configurable explanation depths that adapt to individual case complexity, ensuring that these tools serve as trustworthy and efficient assets in routine radiological reporting.

## Supplementary Information

Below is the link to the electronic supplementary material.

Supplementary file1 (PDF 665 kb)
