# Supplementary material for: Examining explainable artificial intelligence in TNM staging with PET-CT: a user-centred observation study
Source: Radiol Med. 2026 Jul 13;131(9):1601–12. doi: 10.1007/s11547-026-02226-9 (PMC13658394; doi:10.1007/s11547-026-02226-9)
Supplement: Supplementary file 1 [file 11547_2026_2226_MOESM1_ESM.pdf]

Examining Explainable Artificial Intelligence in TNM Staging with PET-CT: A User-Centred Observation Study

Charlie Baskerville[1,2], Julien M. Y. Willaime[2],Vineet Prakash[1,3,4], Tamir Ali[5], Shaheel Bhuva[6], Gordon Ellul[7], Russell Frood[8,9], Sachin Kamat[10], Sudeshna Maitra[3], David Rosewarne[11], Andrew Scarsbrook[8,9], Peter D. Strouhal[4], Amir Zarei[8], Kevin Wells[1]

[1] Centre for Vision, Speech and Signal Processing, The University of Surrey, Guildford, United Kingdom
[2] Mirada Medical Ltd., Oxford, United Kingdom
[3] Royal Surrey NHS Foundation Trust, Guildford, United Kingdom
[4] Alliance Medical Ltd., Warwick, United Kingdom
[5] The Newcastle Upon Tyne Hospitals NHS Foundation Trust, Newcastle upon Tyne, United Kingdom
[6] Oxford University Hospitals NHS Foundation Trust, Oxford, United Kingdom
[7] East Kent Hospitals University NHS Foundation Trust, Canterbury, United Kingdom
[8] Leeds Teaching Hospitals NHS Trust, Leeds, United Kingdom
[9] Faculty of Medicine and Health, University of Leeds, Leeds, United Kingdom
[10] King's College London NHS Foundation Trust, London, United Kingdom
[11] The Royal Wolverhampton NHS Trust, Wolverhampton, United Kingdom

Corresponding Author: Charlie Baskerville (c.baskerville@surrey.ac.uk)

ORCID iD: 0000-0003-1005-2675

February 16, 2026

Supplementary Information

1. Interview Schedule

Post-Task Interview Schedule

1. Do you think the explanation given is plausible?
2. Discuss the quality of this type of explanation.
3. To what extent does this type of explanation provide useful information that allows you to agree or disagree with the staging recommendation? Likert Scale 1-5 (1 not useful, 5 very useful).
    a. Please explain your answer.
4. Using the explanation, can you identify any potential limitations of the AI?
5. Did the addition of the explanation affect your confidence in the AI-system?
    a. On a scale of 1-5 how much did it affect your confidence? (1 no effect, 5 greatly affected)
6. Compared with the black-box system, how was your reporting experience different when using this explainable system?
7. If it was available to you, how likely would you be to adopt a decision support system with this explanation type into your clinical practice compared with the black-box system? 1-5 (1 – a lot less likely, 3 – no change, 5 – a lot more likely)
    a. Please explain your answer.

Final Interview Schedule

8. Could you rank the explanation types?
9. Have your perceptions of adopting AI into clinical practice changed over the course of this session?

2. Mock AI Staging Explanations

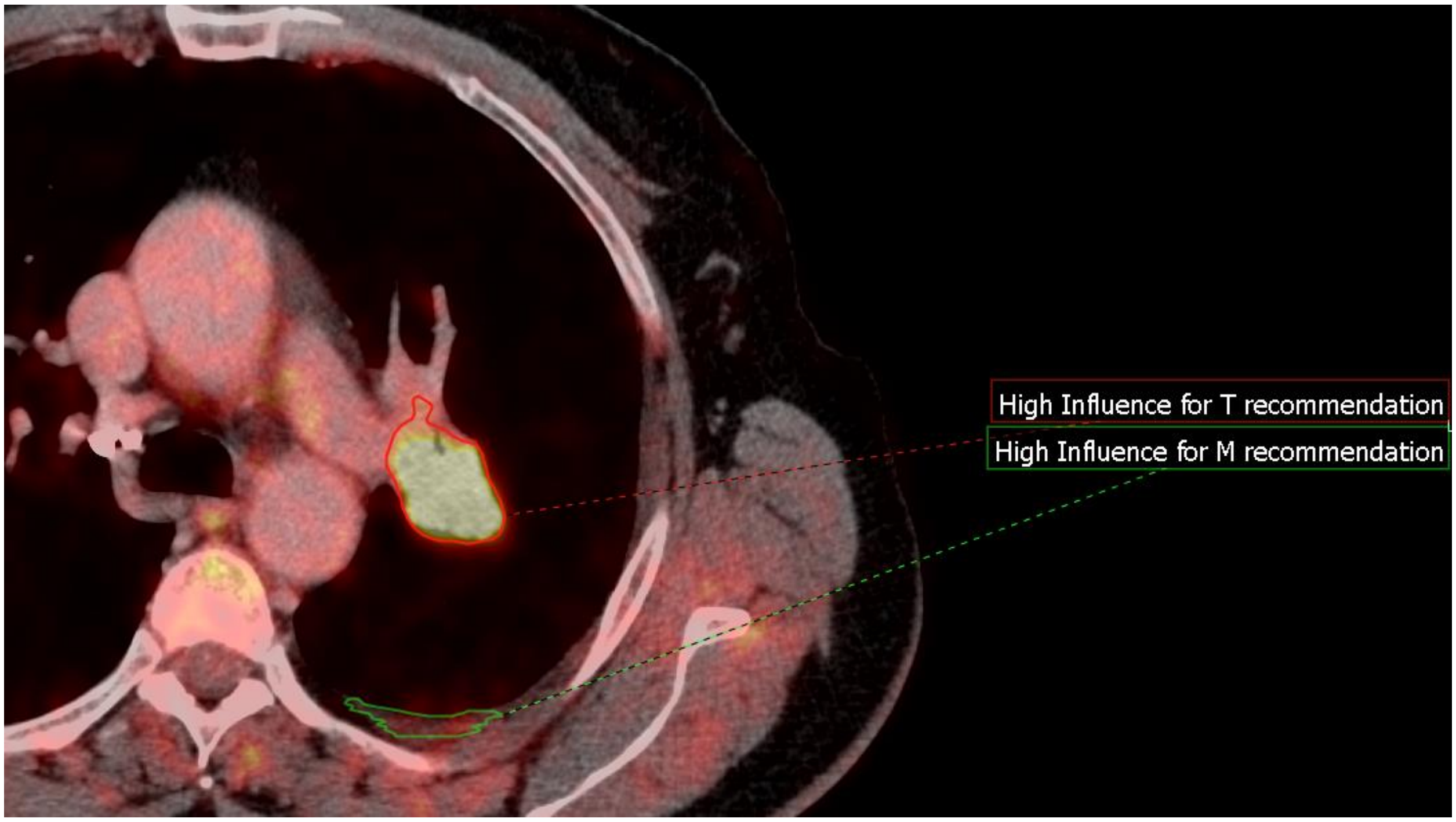

Fig. 1 Example of an AI staging explanation using the 'Input Feature Attribution' interpretability level. Areas of high importance for the decision-making process for T, N and M were shown contoured on the images

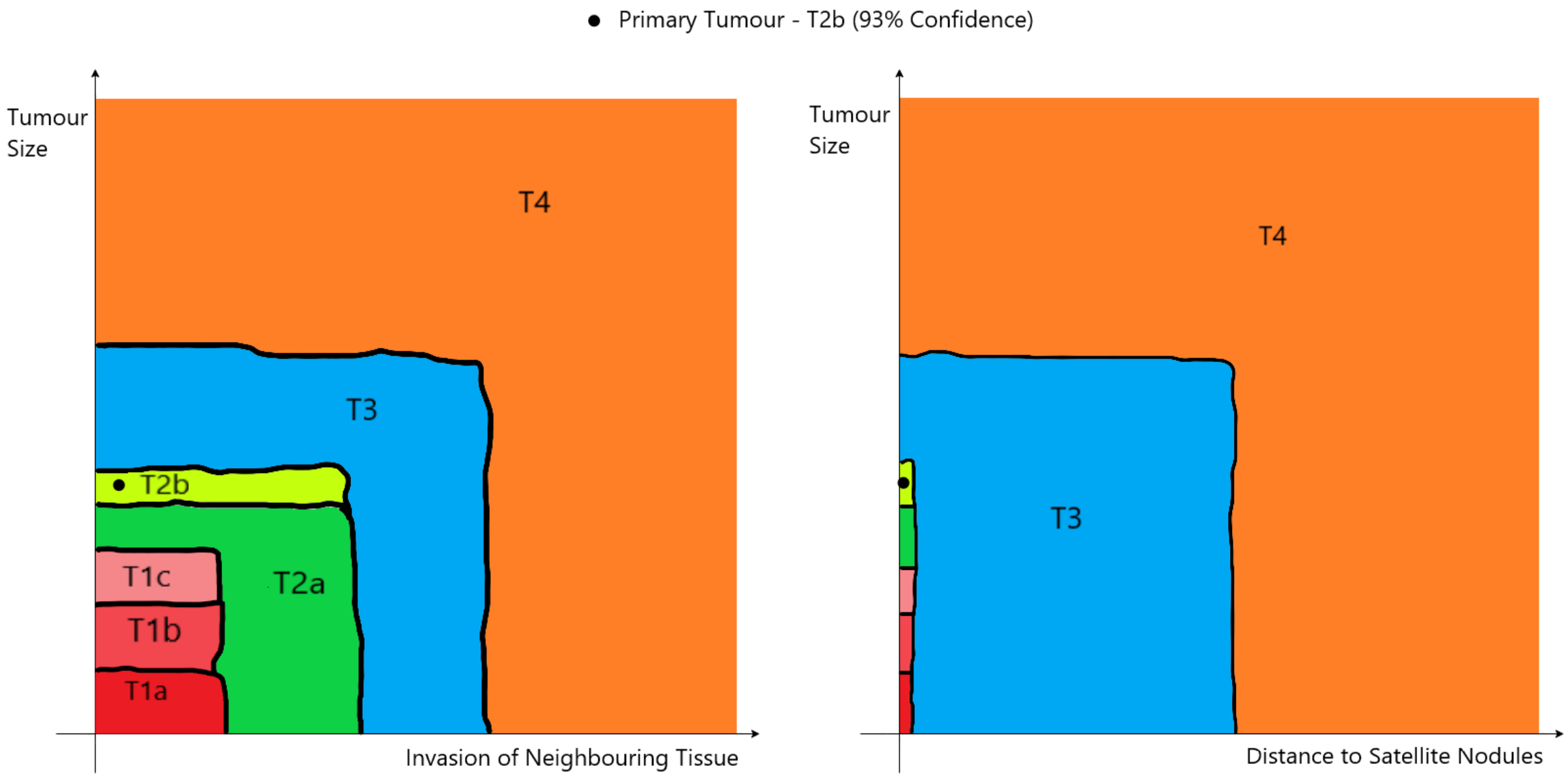

Fig. 2 Example of an AI staging explanation using the 'High-Level Concepts' interpretability level for the T recommendation. This plot represents the AI's decision boundaries with respect to features found to be discriminative during model training, with the black dot representing where a particular case lies within the decision boundary landscape

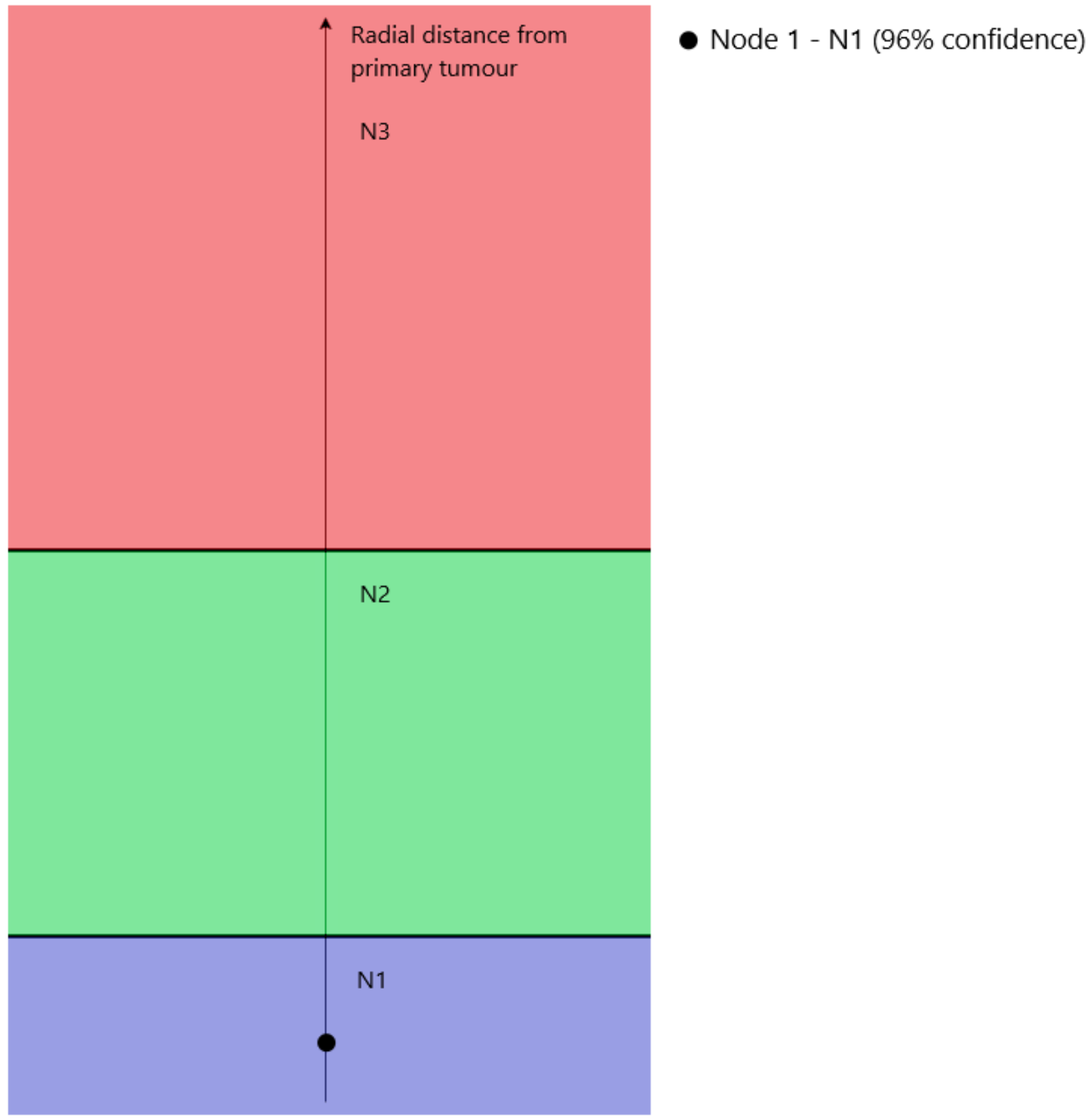

Fig. 3 Example of an AI staging explanation using the 'High-Level Concepts' interpretability level for the node recommendation. This plot represents the AI's decision boundaries with respect to features found to be discriminative during model training. The black dot represents where a particular case lies within the decision boundary decision layers

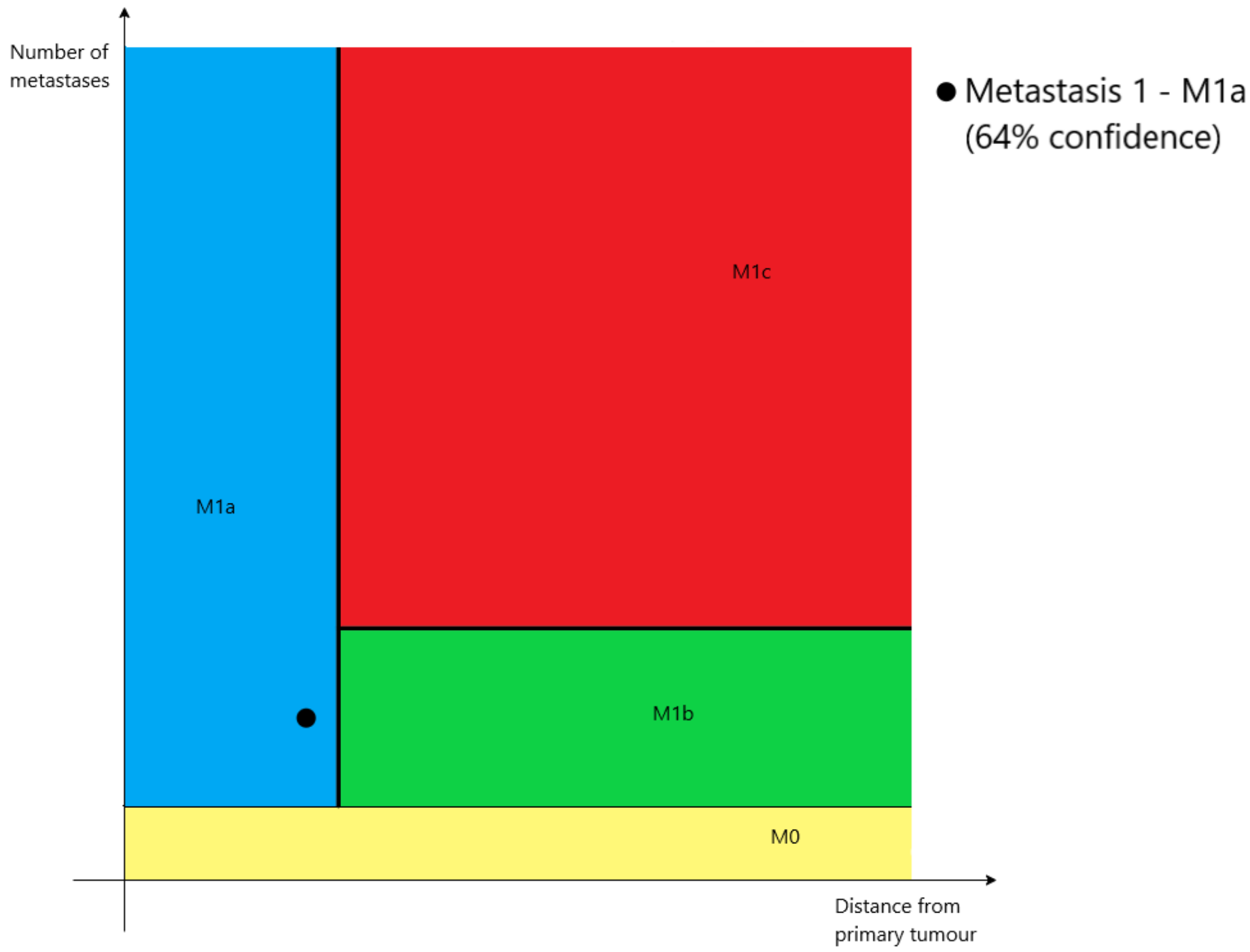

Fig. 4 An AI staging explanation using the 'High-Level Concepts' interpretability level for the M recommendation. This plot represents the AI's decision boundaries with respect to features found to be discriminative during model training. In this exemplar case, the black dot indicates the XAI-CDSS has classified this patient to have M1a disease with 64% confidence

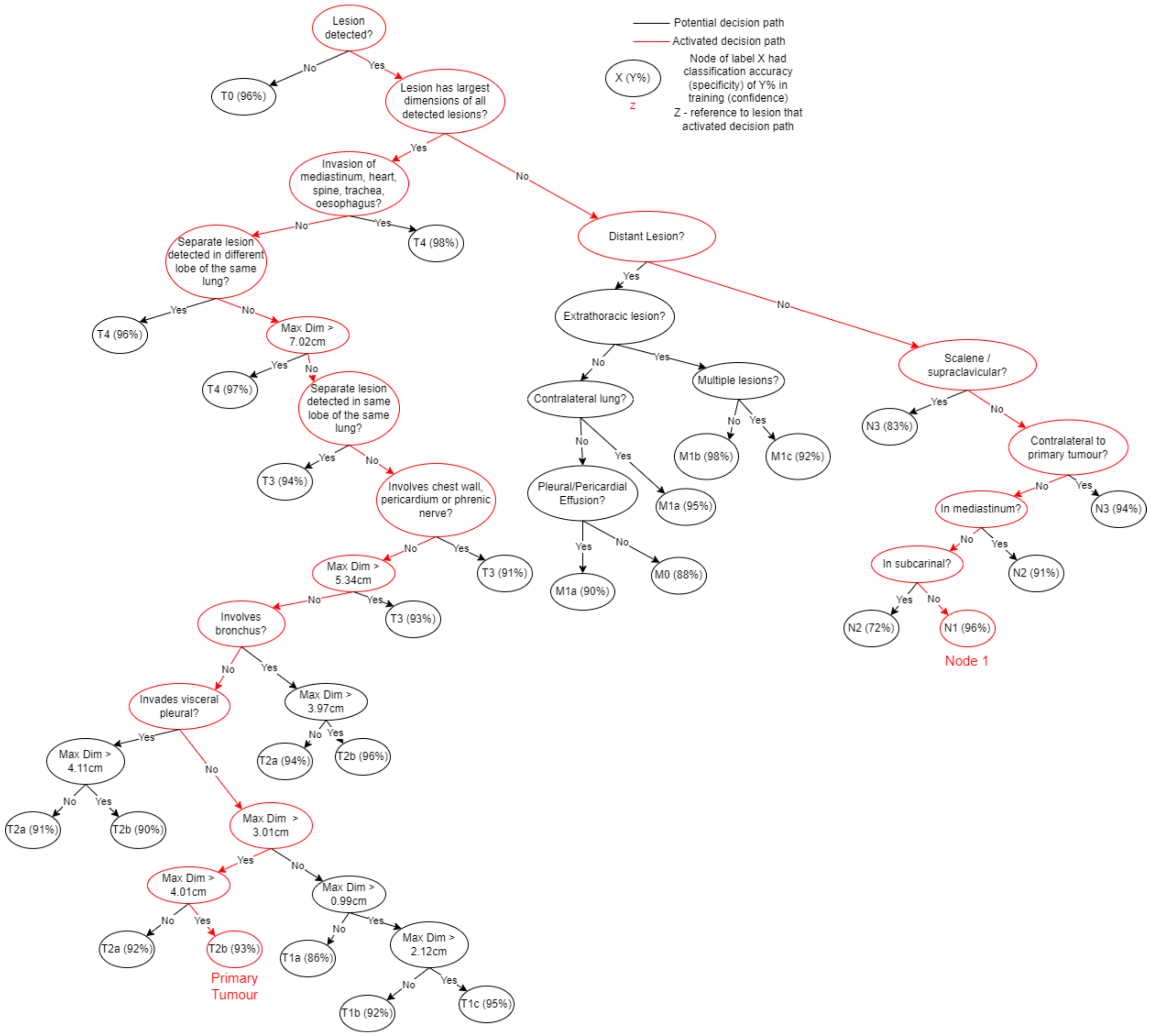

Fig. 5 A staging explanation using the 'Global Algorithmic Transparency' interpretability level for the T, N and M staging recommendation. The full AI decision-making process is mapped out as a decision tree

3. Results – Frequency Distributions

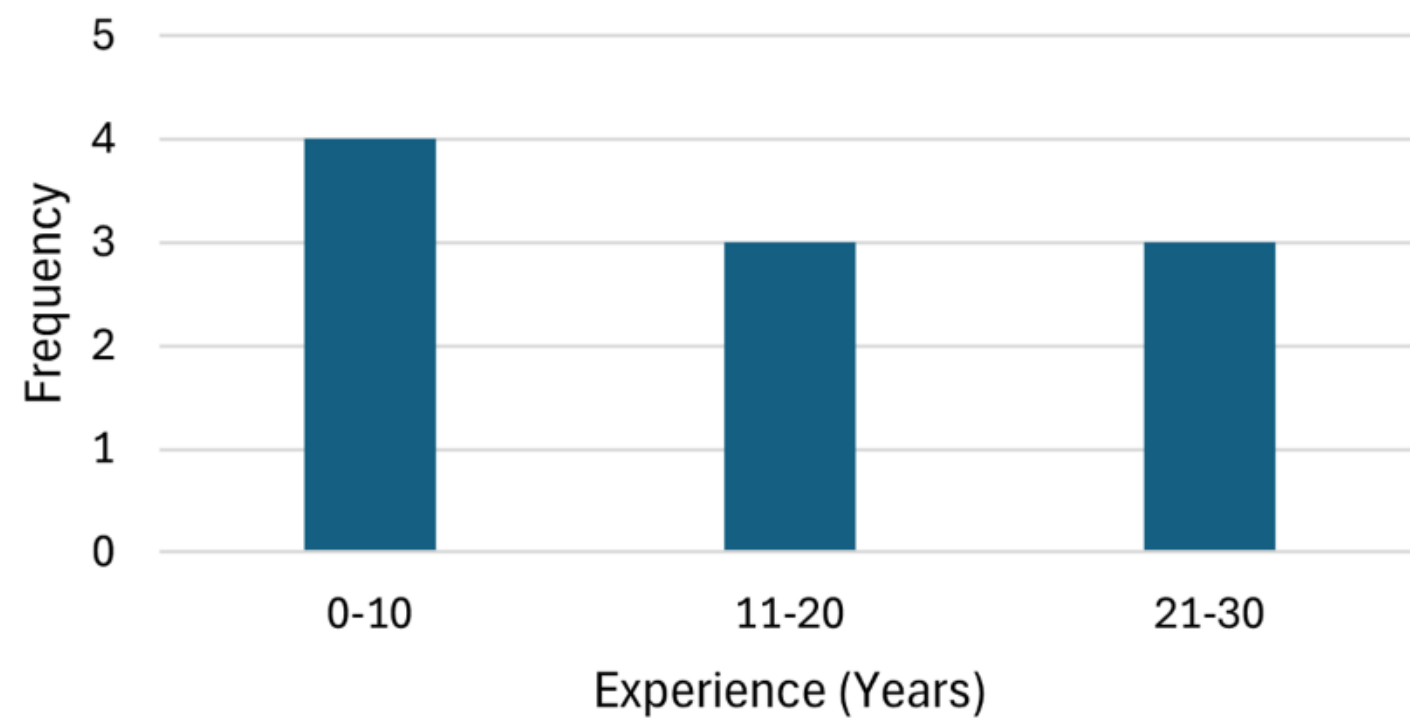

4.

Fig. 6 Frequency distribution chart of radiological experience of the participant cohort

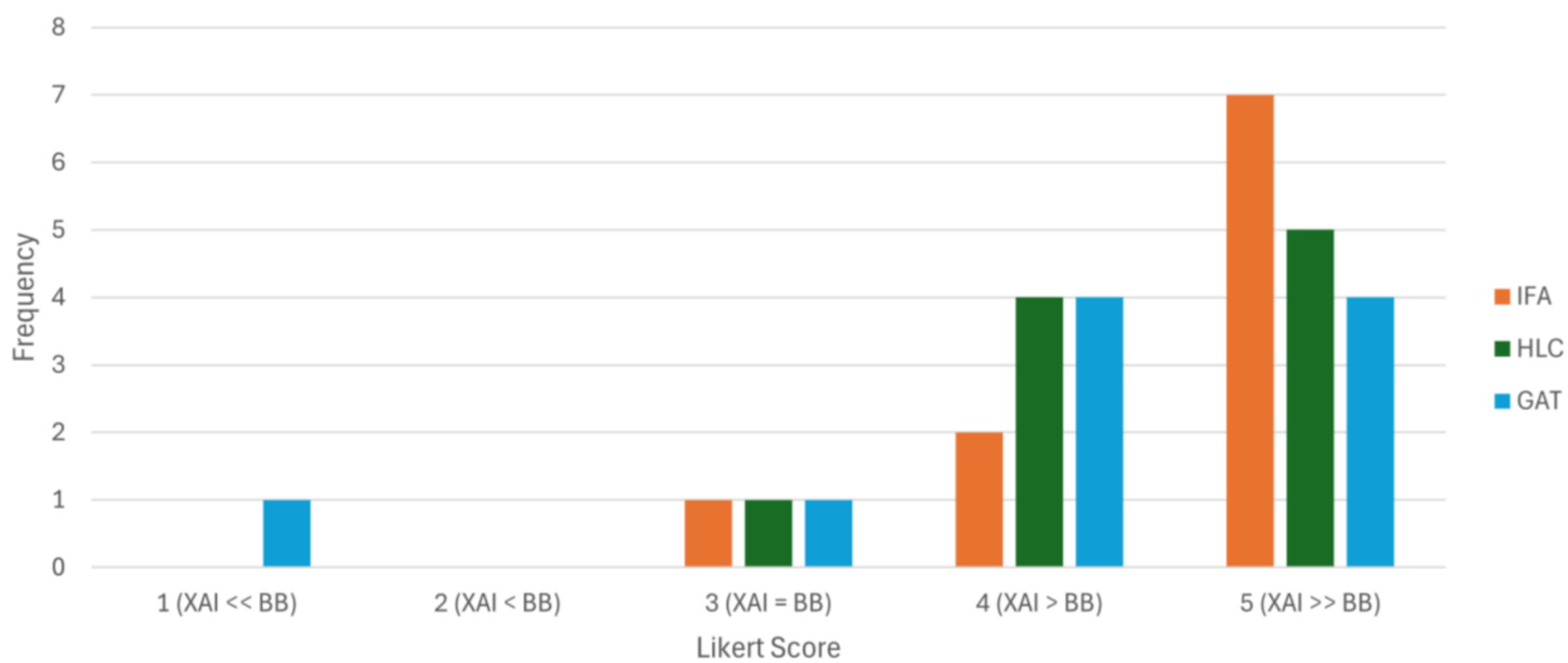

Fig. 7 Adoption likelihood of XAI systems compared with a black-box system (BB) for lung cancer staging (H1 from TABLE 3). Likert score ranged from: 1 - A lot less likely to adopt XAI than the BB (XAI << BB) to 5 – a lot more likely to adopt XAI than the BB (XAI >> BB), with 3 as the neutral response. Input feature attribution (IFA), high-level concepts (HLC) and global algorithmic transparency (GAT). This demonstrates a desire for greater explanation compared to a black-box approach.

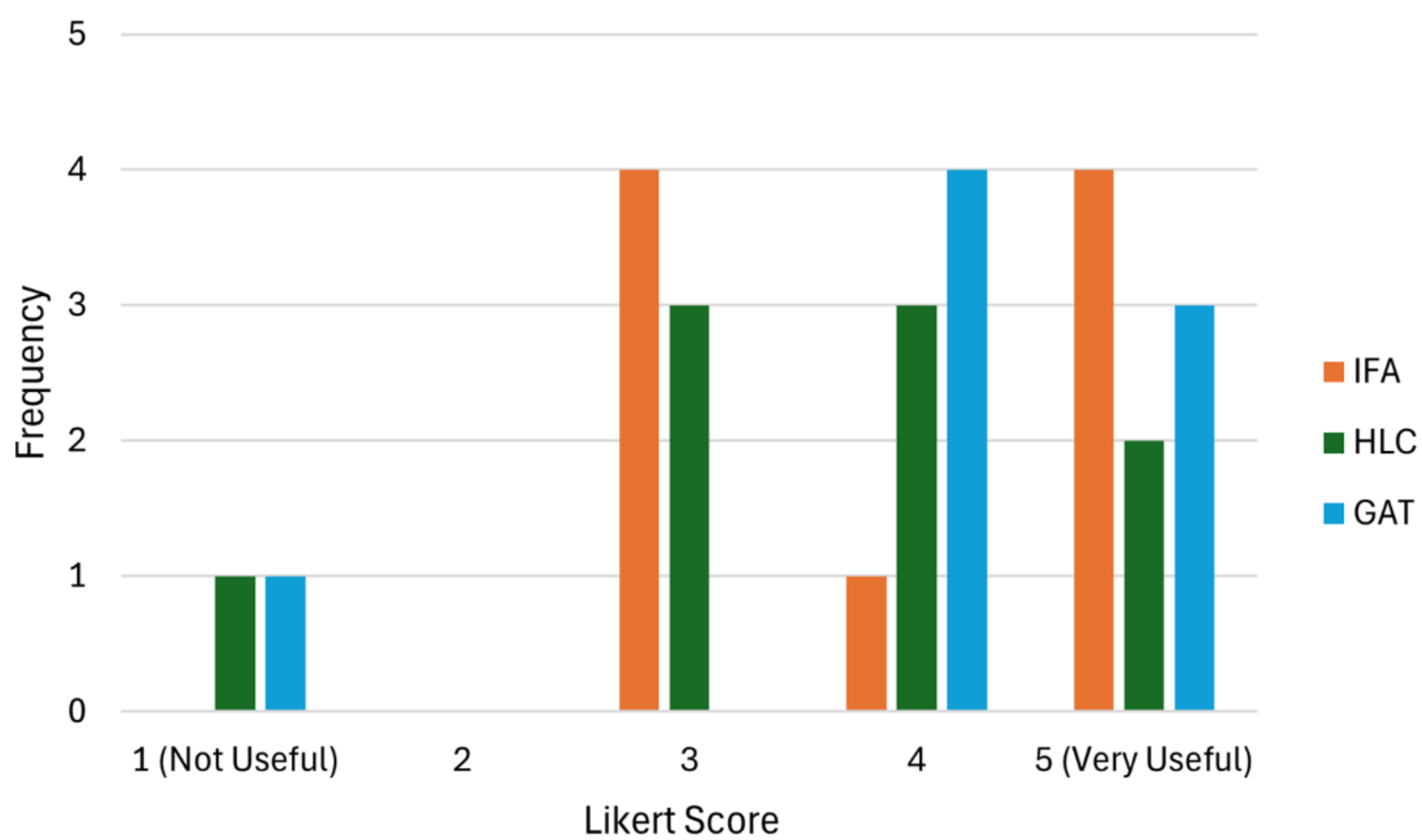

Fig. 8 Frequency distribution chart of Likert scale responses in explanation usefulness evaluation (H2 from Table 2) for input feature attribution (IFA), high-level concepts (HLC) and global algorithmic transparency (GAT). This demonstrates a preference for GAT over other explanation types

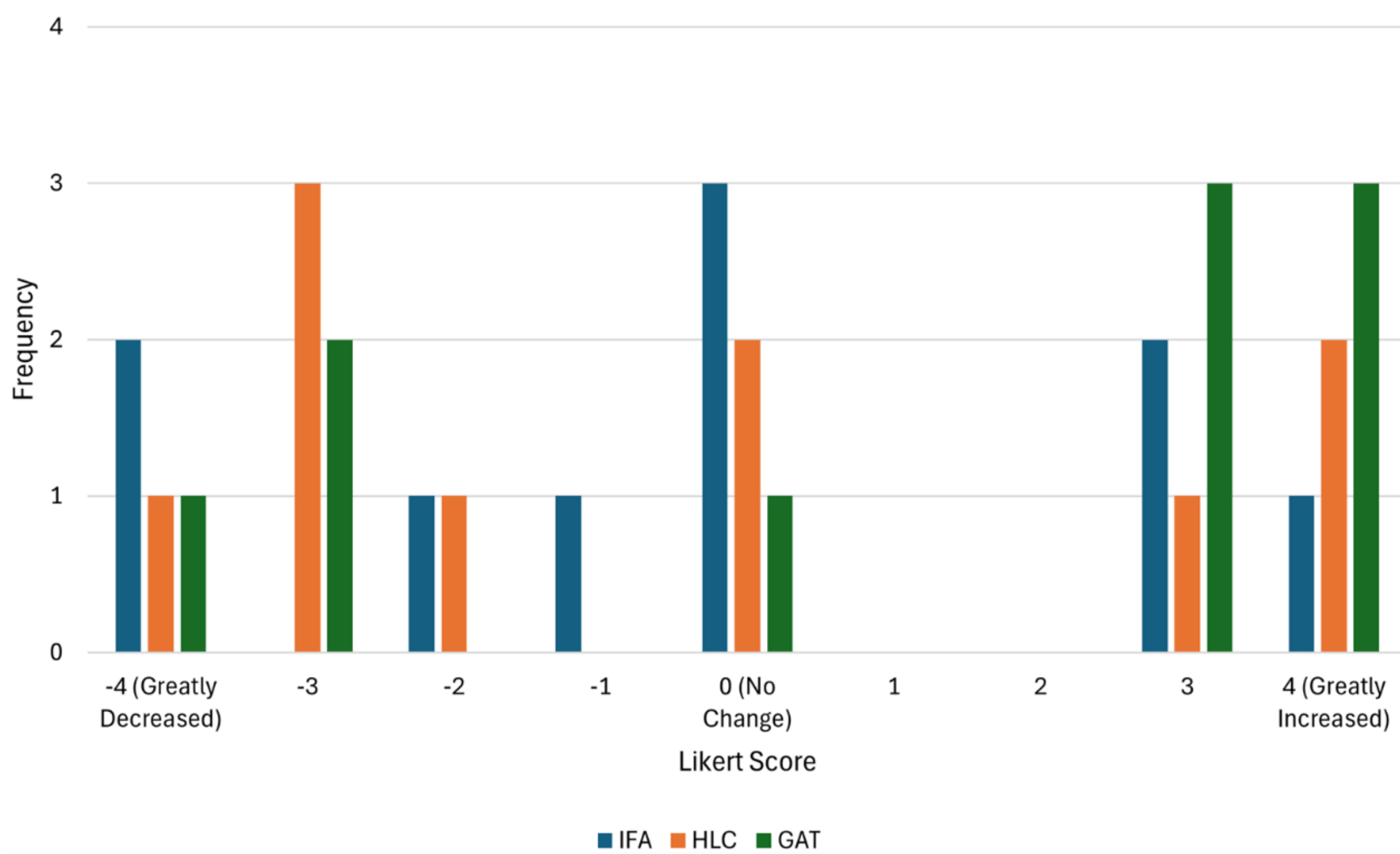

Fig. 9 Frequency distribution chart of Likert score responses about how explanations impacted radiologists' confidence in the AI system for input feature attribution (IFA), high-level concepts (HLC) and global algorithmic transparency (GAT)

4. LLM-Assisted Thematic Analysis

Thematic analysis was performed by CB in collaboration with Anthropic's Claude 3.5 Sonnet. CB conducted interviews, verified and analysed interview transcripts to extract key themes, listed in Table 1.

Table 1 Categories and themes extracted from the semi-structured interviews. Also provided is the definition for the theme which was given to Claude 3.5-sonnet in the qualitative analysis

Category	Theme	Definition
**Trust**	Trust	The participant has confidence the system will perform effectively and reliably in a real-world scenario
	AI-System Confidence	The participant has confidence the system will perform effectively for the desired task
**Explanation Quality**	Explanation Usefulness	The explanation provided useful information to the participant for collaborating with the AI system
	Explanation Depth	The level of detail given in the explanation about the AI's decision-making process
	Error Detection	The explanation provided the radiologist with the ability to identify errors made by the AI, either with respect to a single case or the general decision-making process
**Clinical Value**	Clinical Relevance	The content of explanation is specific to the clinical context of the task
	Clinical Integration	The AI system seamlessly fits the current clinical workflow and how the explanation aligns with radiological practices
	Ease-of-Use	How easy the explanation is to interpret and understand is with respect to information length, content, depth and format
	Decision Support Value	How valuable the explanation content is in the context of the clinical decision
	Human-AI Collaboration	How the explanation facilitates collaboration between the radiologist and AI system

Along with the interview transcripts, an initial prompt was given to Claude:

> *"I conducted an experiment which tested different forms of explainable AI in an AI-based clinical decision support system (AI-CDSS). The decision support system produced TNM staging recommendations for lung cancer patients from whole-body PET-CT scans. The radiologists gave an initial stage for the patient and then were shown the staging recommendation from the AI-CDSS. After this, they were shown an explanation of the AI-CDSS recommendation. The type of explanation varied from case to case. After each case, the radiologist would be asked a set of questions comparing the type of explanation to a black-box system (provided only a stage recommendation and a confidence score for T, N and M).*
>
> *The three types of explanation were:*
>
> *1. Input Feature Attribution - Regions of the scans that were important or influential in the AI's decision-making process were contoured.*
>
> *2. High-Level Concepts - Multi-dimensional plots which showed the decision boundaries of the model with respect to features found to be discriminative in the model training process. There was a plot for each T, N and M recommendations and the patient case was plotted on each. The regions that were classified by the model were also contoured on the scans for reference.*
>
> *3. Global Algorithmic Transparency - The complete AI decision-making process represented by a decision tree. The edges and nodes that were activated by regions detected in the scans were displayed in red, all other decision paths were displayed in black. The detected regions were contoured on the scans for reference.*
>
> *The participants would perform staging on one case per explanation type, in the order listed above. I have collated the transcripts and grouped them by explanation type. There is an additional file with extra questions about their experience level and their comparison of explanation types. I would like you to perform a thematic analysis on each of the explanation types, using the following themes:*
>
> *Trust*
>
> *AI-system confidence*
>
> *Clinical Integration*
>
> *Explanation Usefulness*
>
> *Explanation Depth*
>
> *Clinical Relevance*
>
> *Ease-of-Use*
>
> *Decision Support Value*
>
> *Error Detection*
>
> *Human-AI Collaboration"*

A follow-up prompt was subsequently used to focus and elaborate on each theme:

"Evaluate the theme of [THEME] *which is defined as* [DEFINITION]. *Please support your findings with reasoning and traceable supporting quotes. Ensure the supporting quotes belong to the correct explanation type,*

please specify the participant and question number for each quote. Using multiple quotes from the same participants is acceptable, but please list them separately."

To evaluate themes with respect to interpretability preference, the following prompt was used:

"Please summarise the explanations given for ranking explanation types with respect to preference (found in 'Closing Question.docx'). Please support your findings with reasoning and traceable supporting quotes. Ensure the supporting quotes belong to the correct explanation type, please specify the participant and question number for each quote. Using multiple quotes from the same participants is acceptable, but please list them separately."

Prompts were iteratively refined to optimise response accuracy. This process revealed that providing substantial study context and structuring queries across multiple conversational turns yielded optimal results. Claude occasionally generated inaccurate supporting quotes, such as misattributing statements to incorrect explanation types. When such errors occurred, the human coder re-evaluated the affected theme. All outputs were verified by the human coder, who amended any inaccuracies.
